# Recovery and Renewal of Co-Design Approaches in Health: Protocol for a Realist Synthesis

**DOI:** 10.2196/58318

**Published:** 2024-07-17

**Authors:** Maryam Mallakin, Joe Langley, Gillian Harvey, Sarah Walker, Caylee Raber, Nadia Beyzaei, Luz A Paczka Giorgi, Paul Holyoke, Kate Sellen

**Affiliations:** 1 Health Design Studio OCAD University Toronto, ON Canada; 2 Lab4Living Sheffield Hallam University Sheffield United Kingdom; 3 Design Health Research Innovation Lab University of Alberta Edmonton, AB Canada; 4 CoLab for Community and Behavioral Health Policy University of Washington Seattle, WA United States; 5 Health Design Lab Emily Carr University of Art and Design Vancouver, BC Canada; 6 SE Research Center SE Health Toronto, ON Canada

**Keywords:** participatory design, co-design, realist synthesis, equity, diversity, and inclusion, EDI, community of practice, online co-design, virtual platform, internet research ethics, health communication, health design, COVID-19, virtual health, digital health

## Abstract

**Background:**

The COVID-19 pandemic significantly transformed the landscape of work and collaboration, impacting design research methodologies and techniques. Co-design approaches have been both negatively and positively affected by the pandemic, prompting a need to investigate and understand the extent of these impacts, changes, and adaptations, specifically in the health sector. Despite the challenges that the pandemic imposed on conducting co-design and related projects, it also encouraged a re-evaluation of co-design practices, leading to innovative solutions and techniques. Designers and researchers have explored alternative ways to engage stakeholders and end users, leveraging digital workshops and participatory digital platforms. These adaptations have the potential to enhance inclusivity, allowing for a wider range of individuals to contribute their perspectives and insights through co-design and thus contribute to healthcare change.

**Objective:**

This study aims to explore the impacts of the pandemic on co-design and related practices, focusing on co-design practices in healthcare that have been gained, adapted, or enhanced, with a specific focus on issues of equity, diversity, and inclusion.

**Methods:**

The study uses a realist synthesis methodology to identify and analyze the effects of the pandemic on co-design approaches in health, drawing on a range of sources including first-person experiences, gray literature, and academic literature. A community of practice in co-design in health will be engaged to support this process.

**Results:**

By examining the experiences and insights of professionals, practitioners, and communities who were actively involved in co-design and have navigated the challenges and opportunities of the pandemic, we can gain a deeper understanding of the strategies, tools, and techniques that have facilitated effective co-design during the pandemic, contributing to building resilience and capacity in co-design in health beyond the pandemic.

**Conclusions:**

By involving community partners, community of practice (research), and design practitioners, we expect closer proximity to practice with capacity building occurring through the realist process, thus enabling rapid adoption and refinement of new techniques or insights that emerge. Ultimately, this research will contribute to the advancement of co-design methodologies and inform the future of co-design in health.

**International Registered Report Identifier (IRRID):**

DERR1-10.2196/58318

## Introduction

### Background

Over the past decade, co-design as an approach for health system and service improvement has been rapidly adopted as a stakeholder-engaged process that promotes inclusivity and facilitates change. Co-design is a design approach that involves communities in collaboratively addressing health challenges while enhancing capacity [1]. In recent years, the adoption of co-design techniques in the health care sector has significantly increased, representing a crucial advancement toward integrating real-life experiences, incorporating unconventional knowledge, community-based decision-making, and shared responsibility, in the development and implementation of more equitable health care solutions [2-5]. Drawing on participatory and action research [6], co-design relies on the active involvement of all stakeholders, specifically including individuals with lived experience in conjunction with health care providers, and conventionally incorporates in-person activities. Unfortunately, the pandemic abruptly disrupted the opportunity for in-person engagement.

The COVID-19 pandemic significantly transformed the ways and landscape of work and collaboration, including having a profound impact on design and research methodologies. Despite the challenges that the pandemic imposed in conducting co-design projects in person, designers and researchers have explored and experimented with new ways to engage stakeholders and end users, leveraging digital workshops and participatory digital platforms. These adaptations have the potential to enhance inclusivity, allowing for a wider range of individuals to contribute their perspectives and insights. Rapid adjustments were made to participatory practices and techniques, but co-design, especially for marginalized and equity-seeking groups, experienced setbacks such as limitations, delays, and some loss of effectiveness [7]. Thus, co-design approaches have been both negatively and positively affected by the pandemic, prompting a need to investigate and understand the extent of these impacts, changes, and adaptations, specifically in the health sector.

The “Recovery and Renewal of Participation in Healthcare Change” project aims to enhance capacity and resilience in co-design within and for the health care sector. Its primary objective is to capture the experiences and adaptations that emerged in co-design projects during the pandemic. By examining the evolution of co-design practices, the project seeks to understand how these practices engaged or disengaged communities, and specifically marginalized communities, in health service research, delivery, and improvement throughout and beyond the pandemic. To ensure that the lessons learned during the pandemic are available to shape future co-design initiatives, this project also has a goal to establish a community of practice (CoP) in advanced co-design for health and social care. This community will serve as a platform for collaboration, learning, and knowledge sharing among stakeholders involved in co-design initiatives, highlighting, sharing, and codeveloping new guidance on techniques that can endure beyond the pandemic. This guidance will provide practical recommendations and strategies for implementing co-design methodologies in health care settings, ensuring their sustainability and ongoing impact.

The project’s budget also includes provisions for participant involvement, feedback reflection sessions, and representation of Black, Indigenous, and people of color and equity-seeking community members. The project aims to use case studies from different research teams and community partners, focusing on marginalized groups experiencing health or social status stigma. The engagement mechanisms will be flexible and inclusive to ensure diverse perspectives are included.

### Project Objectives

The “Recovery and Renewal of Participation in Healthcare Change” project aims to enhance co-design capacity in health care by learning from pandemic co-design experiences. It examines how co-design practices affected communities, specifically marginalized communities in health research and delivery. It also establishes a health care co-design CoP for ongoing collaboration and knowledge sharing around co-design adaptations and emerging practices. The project’s main goal is to create practical guidance for effective co-design techniques in health care beyond the pandemic.

The pandemic has led to significant changes in co-design techniques, providing a unique opportunity to address fundamental concepts integral to participation and change [8], such as power relations, equity, and inclusion, including gender-related issues. To this end, this project aims to explore co-design practices that have been gained, adapted or enhanced, and adopted with specific emphasis on the engagement of marginalized, vulnerable, and equity-seeking groups. This will include a specific exploration of topics related to equity diversity and inclusion that intersect with principles of participation and co-design (refined as part of the realist approach) [9-13]

### Research Questions

The project’s research questions are as follows, with a special focus on issues of equity, diversity, and inclusion (EDI): (1) how were co-design practices changed or adapted in what circumstances to give rise to successful or high-quality outcomes for participation? (2) What are the underlying mechanisms that explain how adapted co-design practices worked to enable participation in different contexts? (3) What generalized and context-specific recommendations emerge from these experiences to inform new approaches to postpandemic co-design practice particularly considering EDI?

## Methods

### Study Design

The project uses a realist synthesis approach that engages the CoP as well as our community partners [14,15] together with a design research approach [16].

The project consists of three main phases: (1) planning and detailing the research plan and materials including activating the CoP through existing networks and venues; (2) data collection, analysis, and initial synthesis, CoP involvement in candidate guidelines and principles; and (3) translation of this work into resources, such as case study or demonstration project materials, and detailing of techniques for training and sharing.

A realist approach recognizes how “activities are brought about by the underlying mechanisms constituted by people’s reasoning, and the resources they can summon in a particular context” [15]. This reflects the state of co-design research in health where research practice is emerging through the pandemic and community partners’ experience of participation is highly contextual. While realist approaches will be able to give us an understanding of what works in different contexts for different people, designing will enable us to apply this understanding in new and better ways of working [16,17]. Design practice extends the project methodology from creating knowledge (realist synthesis) to applying knowledge and learning through that process (research through design by demonstration projects) through the CoP supported by this project, and capacity building in new co-design methods. Both realist and design approaches accept a plurality of sources of evidence and perspective, both are iterative, and both are participatory, aligning with the project’s conceptual underpinning in participatory practice and constructivist epistemology, emphasizing the importance of involving multiple stakeholders and recognizing the cocreation of knowledge [15-17].

The study will use multiple sources of evidence, including academic and gray literature, blogs, expert opinions, and lived experiences of participants and community partner representatives. Within the frame of realist approaches, the focus is on the relevancy of the data and evidence using specific criteria generated in collaboration with a CoP rather than screening various types of data through traditional scientific credibility or validity standards, using specific criteria in collaboration with partners. The research direction will be informed by a CoP consisting of health researchers, design researchers, and co-design research contributors.

### Ethical Considerations

The project was approved by the Ontario College of Arts and Design University Research Ethics Board (#102248) to involve human participants. Incentives were only offered to participants who requested them if meeting the EDI criteria.

Individuals signed a consent form to participate in workshops and interviews. All the data gathered throughout these research activities were deidentified for analysis. Membership of the CoP had to be self-initiated so no consent form was required, it is primarily a vehicle for knowledge mobilization and is not a data-gathering instrument.

### CoP Overview

The project team will be closely involved in planning the research plan and materials for the recruitment process, data collection, analysis, synthesis, and translation of the findings into candidate guidelines. An online CoP will be established as a collaborative platform, inviting individuals to share personal knowledge, experiences, and best practices related to co-design for health. This platform will foster a participatory and inclusive environment for engagement.

We will assemble design researchers and practitioners from diverse geographies and equity-seeking groups who have shared adaptations in their practice. We aim to recruit from or through this developing CoP. The composition of the CoP will be purposely built as diverse and equitable, a deliberately diverse pool of collaborators, comprising co-design practitioners, co-design partners (from previous projects), co-design early adopters from other fields, and community members who were involved in co-design projects during the pandemic.

EDI considerations underpin the conceptual orientation of the project from the aim and objectives to our engagement with the CoP, community partners, and the theoretical orientation of participatory and co-design approaches whose intent is engagement in shared decision-making and change inclusive of underrepresented groups.

We will take a purposive sampling approach in our research methods to address EDI considerations in the structuring of the data collection. In this way, EDI considerations will be activated in part through the CoP. The research team is dedicated to respecting and valuing the contributions of all stakeholders, including co-design communities, community partners, researchers, designers, and patient representatives. They will ensure equity and inclusivity in participant selection and data analysis, obtain informed consent, and protect confidentiality and privacy. The study will adhere to human research ethics guidelines.

The realist methods will produce specific context, mechanism, and outcomes statements (CMOs), which will serve as design criteria in phase 3 of the project (design research and capacity building phase). The intent is to identify the underlying mechanisms that explain how adaptations to co-design work in different contexts, and to generate context-specific recommendations for how co-design can be implemented postpandemic or during pandemic conditions. To conduct a realist synthesis, the process will be started by formulating a theory of change, which outlines the key assumptions about how co-design was expected to be impacted by the pandemic, what contexts saw what changes and impacts, and what the outcomes were of these co-design experiences. Further, the team will systematically search for evidence to test the theory of change, using various sources such as published and unpublished studies, gray literature, and first-hand experiences of co-design. The collected evidence will be subjected to analysis using a realist approach, entailing the identification of patterns and trends in the data to develop and refine the theory of change [18,19].

The realist synthesis process consists of the following key stages:

#### Stage 1: Formulating Research Questions

The study’s foundation is built upon well-crafted research questions focused on understanding the impact of the pandemic on co-design with a focus on marginalized communities’ involvement in health projects. These questions guided the investigation and framed this study’s objectives.

#### Stage 2: EDI Framework

Drawing from existing research and insights provided by diverse teams involved in this study, an EDI framework will be carefully developed to support the application of the theory of change articulated in stage 1. This framework acts as a lens through which the research data will be analyzed, ensuring sensitivity to issues of EDI throughout this study.

#### Stage 3: Data Collection 1

#### Realist Review

A comprehensive realist review will be conducted to explore information about co-design during the pandemic, including adapting co-design techniques, challenges, limitations, and emerging practices. Additionally, we aim to address the initial study question of identifying effective mechanisms for remote and distanced co-design to ensure the engagement of the most impacted communities and marginalized populations in designing and implementing health solutions. The realist review process will commence with the collaborative creation of the initial reference set of articles by the project team. This will be achieved by using relevant keywords such as “co-design,” “COVID-19,” “remote,” “distanced,” “virtual,” “hybrid,” and exploring author reference lists in sources such as Google, Google Scholar, national or international design journals, and digital libraries like the Ontario College of Art and Design University library databases, which include CEL (University of Ljubljana School of Economics and Business), Academic OneFile (Gale), JSTOR (ITHAKA), and Literature Resource Center (Gale). To expand the scope of literature research, artificial intelligence literature search tools such as OK-maps (OkMap) and Litmaps (Litmap Ltd) will also be used. Additionally, gray literature searches will encompass social media platforms, including blogs, facilitated Instagram (Meta) and Twitter (X Corp) discussion posts, and threads related to co-design. Moreover, information from the co-design CoP, news, and co-design events such as workshops will be explored to further enrich the realist review process. This review will provide essential context and establish the groundwork for this study.

#### Workshops

Multiple online workshops will be organized, recruiting participants actively involved in or planning or managing co-design efforts within health projects during the pandemic. These workshops are designed to accommodate participants across 5 different time zones (EST, MST, PST, GMT, and Australian Eastern Daylight Time), ensuring inclusivity and widespread engagement. The primary objective of these workshops is to create a dynamic platform for knowledge exchange, enabling participants to share insights, experiences, and adaptations related to co-design during the pandemic. To facilitate seamless communication and collaboration, Zoom (Zoom Video Communications, Qumu Corporation) will be used as the virtual meeting platform, while Miro will serve as the collaborative virtual whiteboard. The workshop activities will be thoughtfully designed, drawing upon co-design and EDI principles identified from prior research [20-22]. By incorporating these principles, the workshops aim to foster an environment that encourages open dialogue and valuable exchange of ideas. Through these interactive sessions, participants will have the opportunity to explore the challenges, questions, successes, and failures encountered in co-design practices through the pandemic. The collective knowledge gained from these workshops will contribute to a deeper understanding of the adaptations and gains made in co-design during the pandemic. This will be used to develop initial CMOs’ analyses that explain the achieved outcomes within different contexts and by various mechanisms [18].

#### Stage 4: Synthesis and Data Collection 2

#### Overview

The study will use an inductive coding approach, beginning with the EDI framework to support the interrogation of preliminary theories. A further round of analysis will be used to abstract demi-regularities in the data, which will be input to developing initial CMOs. To analyze the resources gathered for our research (realist review and workshops), we will use ATLAS.ti (ATLAS.ti Scientific Software Development GmbH), a qualitative research tool designed for coding and analyzing various types of data such as published papers, transcripts, workshop data, blogs, and social media content.

#### Interviews, Webinars, and Online Survey

To address any potential data gaps identified during the research and gain more insights from relevant stakeholders, a series of interviews and webinars will be conducted. These interactions enrich this study by incorporating a wide range of diverse perspectives. The interviews will provide a valuable opportunity to delve deeper into specific aspects of the research, enabling us to gain deeper insights and gather nuanced information from key participants. Individually, the webinars will foster a collaborative environment where individuals who are involved in co-design practices with various backgrounds and expertise can actively engage in discussions and share their knowledge and experiences related to co-design for health. An online survey will be distributed to test, develop, and build consensus around identified contexts, mechanisms, and outcomes that led to adaptations or led to losses or failures of co-design in health through the pandemic. We are planning to conduct surveys in 2 formats: an initial short survey to assess the initial CMOs during our webinars, followed by a more comprehensive survey at a later stage to validate the final CMOs.

#### Stage 5: Evaluation and Validation Workshops and Survey

The findings of this study will be subjected to evaluation and validation workshops. This process ensures that the final CMOs are credible, and accurately reflect the impact of online co-design on marginalized communities’ involvement and co-design techniques in health projects. We will also use surveys to validate our findings and achieve a consensus regarding the final CMOs. To do this, we will initially distribute concise surveys during CoP webinars to evaluate and test the Middle-range CMOs. Subsequently, we will use the Delphi survey technique which includes a series of iterative questionnaires. It will enable us to validate and establish a shared consensus regarding the final CMOs.

#### Stage 6: Demonstration Project Materials, Training, and Sharing

As an outcome of stages 1-5, this study will contribute valuable insights in the form of case studies, CoP dialogue, webinars, and training opportunities. This will include a refined EDI framework and guidelines to support and enhance resiliency [23] in co-design research and practice through capacity-building activities through the CoP.

## Results

By following the realist synthesis process, the potential outcomes or results of this study will include a comprehensive understanding of how remote and distanced co-design and co-design adaptations can be leveraged effectively for designing and implementing health solutions with a focus on EDI.

The timeline for the project encompasses various stages, including realist review, data collection, data analysis, synthesis and evaluation, design and development of framework and guidelines, development of capacity-building materials, knowledge mobilization activities, and reporting over 3 years.

This project was initially funded in March 2022 and has hosted 7 webinars, 7 workshops, 1 winter school, and 15 interviews as of May 2024. The CoP has been active since October 2022 and has 169 members as of May 2024. Knowledge dissemination will involve journal publications, workshops, and talks across academic contexts to share key findings.

## Discussion

### Principal Findings

By examining the experiences and insights of professionals, practitioners, and communities who were actively involved in co-design, and having navigated the challenges and opportunities of the pandemic, we can gain a deeper understanding of the strategies, tools, and techniques that have facilitated effective co-design during the pandemic. The insights gathered can contribute to building resilience and capacity in co-design in health beyond the pandemic. Furthermore, the involvement of community partners, CoP, and design practitioners is expected to be closer to practice, thus allowing for capacity building, and potentially enabling rapid adoption and refinement of new techniques or insights that emerge.

### Expected Findings

This project will explore and capture how co-design practices, adaptations, and experiences (including failures) emerged during the pandemic. It will also identify resilient practices, new practices that hold promise for enabling co-design in health, and ways in which equity and inclusion can be enhanced.

The potential results of this study will include an in-depth understanding of how co-design adaptations can be leveraged effectively to enhance co-design practices, for designing and implementing health solutions with a focus on EDI. Learnings from this work will then contribute to rapidly advancing co-design practices in health, with a specific focus on issues of EDI, while also providing new guidance on techniques that can enhance resilience while building capacity in co-design research and practices.

### Strengths and Limitations

The project’s key strengths lie in its application of an approach that combines realist synthesis and design approach. This approach empowers the project team to attain the essential knowledge needed for the development of comprehensive guidelines. The realist methodology enables the project team to explore and assess various studies and projects that have used co-design approaches and techniques through the pandemic to identify the fundamental mechanisms and contextual elements that play a key role in effective co-design [16]. Additionally, design approaches permit the project to surpass knowledge creation and use this knowledge to foster innovative and enhanced methods of operation. Moreover, active involvement and collaboration of the CoP and community partners in the project play a pivotal role in ensuring the achievement of the project’s objectives.

Because the project explores emerging practices it may encounter some limitations, including that there might be limited existing research or data available for reference, the rapidly changing nature of the emerging practices makes it challenging to maintain up-to-date findings throughout the project duration.

### Future Directions

We will use a range of various knowledge translation and sharing methods to disseminate our research findings. Our ongoing connection and collaboration with CoP members provide an opportunity to share, exchange, and disseminate our findings within the network and to broader audiences. Input from CoP members and other knowledge users will inform our dissemination strategies and guide the planning of future research initiatives. Additionally, the research team’s strong connections to key journals in this space, such as JMIR, Design for Health, Health Expectations, and the International Journal of Integrated Care, highlight their ability to disseminate their contributions to a broad audience. Moreover, the outcomes of this project will make visible contributions to the evidence base through open-access publication venues, ensuring widespread accessibility for design researchers and health care improvement specialists.

### Conclusions

The outcomes of this project will lead to the creation of a co-design framework and guidelines aimed at enhancing resiliency in co-design practices through an EDI lens. By focusing on EDI issues specifically in our examination of emerging techniques, we expect to contribute to the rapid development of inclusive co-design that may have positive impacts not only on equity-seeking groups but for all future participants. This framework is set to have an impact on communities in health service research, health service delivery, and health care improvement beyond the challenges posed by the pandemic.

## Abbreviations

CMO: context, mechanism, and outcomes statement
CoP: community of practice
EDI: equity, diversity, and inclusion

## Data Availability

The data sets generated during this study are not publicly available due to ethical considerations. However, key findings that surged from the analysis of these data sets are available in the community of practice [24].
